# The Life-Cycle of the HIV-1 Gag–RNA Complex

**DOI:** 10.3390/v8090248

**Published:** 2016-09-10

**Authors:** Elodie Mailler, Serena Bernacchi, Roland Marquet, Jean-Christophe Paillart, Valérie Vivet-Boudou, Redmond P. Smyth

**Affiliations:** Architecture et Réactivité de l’ARN, Université de Strasbourg, IBMC, CNRS, 15 rue René Descartes, 67084 Strasbourg, France; e.mailler@ibmc-cnrs.unistra.fr (E.M.); s.bernacchi@ibmc-cnrs.unistra.fr (S.B.); r.marquet@ibmc-cnrs.unistra.fr (R.M.); jc.paillart@ibmc-cnrs.unistra.fr (J.-C.P.)

**Keywords:** HIV-1, packaging, Gag, genomic RNA, assembly

## Abstract

Human immunodeficiency virus type 1 (HIV-1) replication is a highly regulated process requiring the recruitment of viral and cellular components to the plasma membrane for assembly into infectious particles. This review highlights the recent process of understanding the selection of the genomic RNA (gRNA) by the viral Pr55^Gag^ precursor polyprotein, and the processes leading to its incorporation into viral particles.

## 1. Introduction

Human immunodeficiency virus type 1 (HIV-1) replication is a highly regulated process requiring the recruitment of viral and cellular components to the plasma membrane for assembly into infectious particles. The Pr55^Gag^ precursor polyprotein (Gag) is the minimal component required for HIV-1 assembly. It specifically recruits the viral genomic (gRNA) via its nucleocapsid (NC) domain, and drives viral assembly through the multimerization of its capsid (CA) domain at specific plasma membrane micro-domains targeted by the matrix (MA) domain [1,2,3,4,5]. The RNA genome acts as a scaffold for the multimerization of Gag and Gag–Pol precursors and is a key player in retrovirus assembly [6,7].

**Figure 1:**
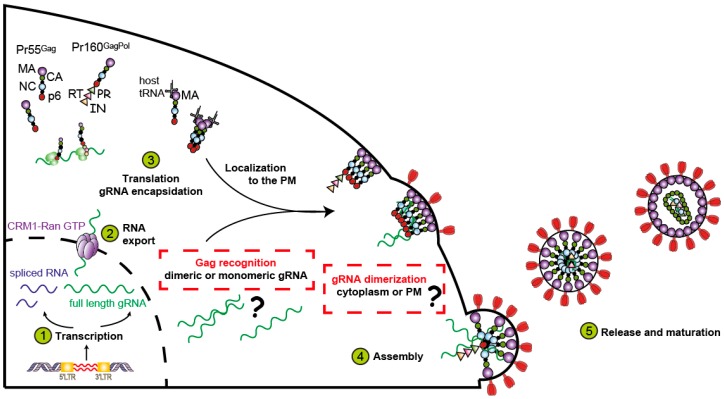
The late phase of retroviral cycle highlighting the dimerization event and the Gag–RNA complex formation. Full-length genomic RNA (gRNA) as well as singly and multi-spliced viral RNAs are produced by the host cell machinery and exported into the cytoplasm. Two copies of genomic RNA (gRNA) are encapsidated inside the newly synthesized viral particle as a dimer. The interaction of Gag with the gRNA in the cytoplasm ensures its specific encapsidation. Targeting of the Gag–RNA complex to the plasma membrane (PM) is promoted by host transfer RNA (tRNA) binding to the Gag matrix (MA) domain. Since the spatio-temporal parameters of these related events remain unclear, this figure depicts the different possibilities. CA: capsid; NC: nucleocapsid; RT: reverse transcriptase; PR: protease; IN: integrase; CRM1: Chromosomal Maintenance 1; LTR: long terminal repeat.

In order to generate an infectious particle, HIV-1 must selectively package two copies of its unspliced positive sense and single-stranded gRNA [8,9]. Genome encapsidation is highly specific, allowing the gRNA to be efficiently selected from a much larger pool of cellular and subgenomic viral RNA species (reviewed in [10,11]). This specificity is achieved through the recognition of *cis*-acting packaging signals by the Gag precursor protein which are also thought to be regulated by RNA conformational switches [12,13,14,15,16]. The major packaging signal comprises the 5′ untranslated region (UTR), the beginning of the Gag coding sequence [17,18,19,20,21,22,23], and may include other regions within the gRNA [24,25,26]. The gRNA is initially selected in the cytoplasm by a limited number of Gag molecules. The Gag–RNA complex then nucleates viral assembly at the plasma membrane. However, the spatio-temporal parameters of gRNA recognition and viral assembly remain incomplete (Figure 1).

This review will summarize our current understanding of the HIV-1 Gag–RNA complex formation leading to its packaging into viral particles.

## 2. Nuclear Export of Genomic RNA and Its Role in Particle Assembly

The HIV-1 replication cycle begins with the transcription of the proviral DNA by the host RNA polymerase II into full-length viral RNA that can subsequently be singly or multi-spliced [27]. This pool of viral RNA must traffic out of the nucleus into the cytoplasm to serve as messenger RNA (mRNA) for translation as well as gRNA for packaging. The host machinery normally prevents the export of single and unspliced viral RNA species, so retroviruses must circumvent this blockade. HIV-1 encodes the Rev protein which interacts with an RNA element called the Rev response element (RRE) and with the Chromosomal Maintenance 1 (CRM1)/RanGTP nuclear export complex [14,28,29,30,31,32,33].

Interestingly, several studies demonstrate a role for the RNA export pathway in HIV-1 assembly. Indeed, replacement of the HIV-1 RRE with the hepatitis B virus post-transcriptional regulatory element caused virus particles to accumulate intracellularly with slower kinetics despite similar levels of Gag expression compared to Rev-dependent Gag [34,35]. This mutant could be rescued in *cis* by replacing the MA domain with another membrane targeting domain, or in *trans* by expressing Gag from a Rev-dependent mRNA [35]. The defect was attributed to altered Gag localization due to inefficient plasma membrane targeting by MA when the viral mRNA is trafficked out of the nucleus by a Rev-independent pathway [35]. Interestingly, a similar relationship between HIV-1 RNA trafficking and productive Gag assembly was earlier reported in murine cells which normally do not support viral replication due, in part, to inefficient Gag plasma membrane targeting [27,36,37]. In this case, efficient budding could be induced upon modifying the gRNA export pathway [38,39], or through mutation of the MA domain to enhance membrane binding [40]. Taken together, these results suggest a strong influence of the nuclear export pathway on downstream cytoplasmic events leading to viral assembly. Of note, it was recently demonstrated that Rev dependent (containing RRE) and Rev independent (containing the Mason-Pfizer monkey virus (MPMV) constitutive transport element (CTE)) RNAs have remarkably different trafficking behaviours when visualized in the cytoplasm [41]. RRE-containing RNAs exhibited “en masse” export into the cytoplasm, consistent with a diffusion based mechanism of transport [41,42], whereas MPMV CTE-containing RNAs clustered to the microtubule-organizing center (MTOC) [41]. This suggests that RNA export elements can impact later stages of the virus life-cycle through their distinct trafficking behaviours in the cytoplasm.

## 3. The Fate of Genomic RNA: Translation versus Packaging

The full length unspliced RNA serves as the mRNA template for Gag and Gag–Pol synthesis, as well as the genome for packaging. The fact that the cellular translation machinery and viral assembly could compete for cytoplasmic utilization of the gRNA implies a need to regulate these processes. Several groups have proposed models for the regulation of gRNA translation and packaging, but the interplay between these processes is poorly understood.

Experiments using inhibitors to block host cell transcription or translation show that simple retroviruses, such as murine leukaemia virus (MLV), segregate unspliced gRNA into two functionally distinct populations: one associated with ribosomes for translation and one for packaging into viral particles [43,44,45]. This seems not to be the case with HIV-1, as similar experiments show that unspliced RNA can serve interchangeably as mRNA for translation and gRNA for packaging [45]. Nevertheless, even with a single pool of gRNA, Gag may still co-translationally package its own mRNA, which would be useful as a mechanism to preferentially package unspliced gRNA over other RNA species. For HIV-2, prior translation is not absolutely necessary for packaging [45], but early reports showed that the Gag precursor can directly binds to its own mRNA enhancing its packaging [46,47]. Similarly, for HIV-1, although translation does not seem to be absolutely required [48], co-translational capture was suggested to enhance gRNA packaging [49,50]. However, co-translational packaging for both HIV-1 [51] and HIV-2 [52] is not universally accepted, as more recent studies showed efficient packaging of gRNA by Gag in *trans*. At least for HIV-1, the packaging signal is known to include the AUG start codon, and it is likely that previous results supporting a co-translational packaging mechanism were instead due to a direct disruption of the packaging signal.

## 4. Selection of the Full-Length Genome by Gag

### 4.1. Gag

**Figure 2:**
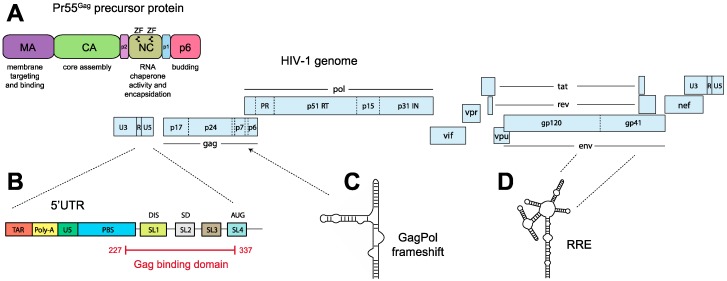
Human immunodeficiency virus type 1 (HIV-1) genome and Gag organization. (**A**) The Pr55^Gag^ protein. Major functions of the different domains are indicated; (**B**) The HIV-1 5′ untranslated region (UTR) contains the major packaging region SL1–SL4. The major Gag binding domain comprises nucleotides 227–237 in the NL43 strain [68]; (**C**) A region overlapping the Gag-Pol frameshift signal and (**D**) the Rev response element (RRE), might also be implicated in packaging. ZF: zinc finger; MA: matrix; CA: capsid; NC: nucleocapsid; p1: spacer domain SP2; p2: spacer domain SP1; TAR: Tat responsive element; Poly-A: polyadenylation signal, U5: unique 5′ region, PBS: primer binding site; SL: stem loop; DIS: dimerization initiation site; SD: splice donor; U3: unique 3′ region; R: repeat region; PR: protease; RT: reverse transcriptase; int: integrase; vif: viral infectivity factor; vpu: viral protein U; vpr: viral protein R; gp: glycoprotein; nef: negative factor.

The specific binding of Gag to the gRNA directs its encapsidation into nascent virus particles. Gag is a polyprotein with four structural domains MA, CA, NC and p6 and two short spacer peptides, SP1 (p2) and SP2 (p1) (Figure 2A). The C-terminal domain of CA contains the protein dimerization interface, whereas the MA domain binds plasma membrane lipids. Membrane binding is directed by a bipartite signal: the myristoyl group at its N-terminus, which facilitates hydrophobic interactions with membranes, and the highly basic region (HBR) at the MA surface, which mediates electrostatic interactions with cellular lipids like phosphatidylinositol-4,5-bisphosphate (PIP_2_) [42,43,53]. MA also interacts with nucleic acids [54,55], and this is now thought to regulate the interaction between Gag and cellular membranes [4,55,56,57]. The NC domain specifically interacts with the gRNA through its two CCHC-type zinc finger motifs [7,58,59]. However, in vitro assays demonstrate that Gag has a higher affinity for the gRNA than the NC domain alone, strongly suggesting that other domains contribute to the Gag-gRNA interaction [13,17,60,61,62]. NC binding to RNA promotes the multimerization of Gag during virus assembly [7], and assembly in vitro can be initiated by a variety of nucleic acids, including short DNA oligonucleotides [58]. Remarkably, NC of HIV-1 Gag can be functionally replaced with a leucine zipper protein-protein interaction domain elegantly demonstrating that RNA per se is not needed for viral assembly, but serves only to promote Gag-Gag multimerization [63,64,65,66,67].

### 4.2. Genomic RNA and the Packaging Signal

HIV-1 packages two copies of its RNA genome into each virion [69]. These genomes are non-covalently linked via a process known as genomic RNA dimerization [9]. The initial interaction between the two genomes occurs in a region called the dimer linkage structure present in the 5′ UTR, also known as stem-loop 1 (SL1) [12,14,70,71,72]. The dimerization process requires a six-nucleotide palindromic sequence present in the SL1 apical loop known as the dimerization initiation site (DIS). For the HIV-1 B strain, this sequence is GC-rich and flanked by two 5′ and one 3′ unpaired nucleotides. It has been shown by mutagenesis and chemical probing that this palindromic sequence forms an intermolecular kissing loop-loop interaction [71,72,73], and it is now well accepted that SL1 plays an important role in multiple steps of the HIV-1 life-cycle, including reverse transcription [74,75], packaging [13,75,76] and recombination [77,78,79]. Interestingly, deletion of SL1 decreases the kinetics of dimer formation [80], but neither viral replication [74] nor RNA packaging [81,82,83] is completely abolished. Furthermore, gRNA extracted from SL1 deleted virions is dimeric with normal thermal stability [80,84], providing strong evidence that additional dimerization sites exist within the HIV-1 genome [81,85]. Even so, it is generally agreed that SL1 is the most important dimerization motif, and the location and function of these putative additional dimerization sites still need to be determined.

The HIV-1 5′ UTR is highly structured and contains a large number of independent domains (Figure 2B) [86,87,88,89,90]. From 5′ to 3′ these are the Tat Responsive Element (TAR) stem-loop required for efficient transcription of the viral RNA; the poly-A hairpin which contains a repressed polyadenylation signal; the primer binding site (PBS) for initiation of reverse transcription. Downstream of the PBS, the packaging signal, or Psi region, folds into four stem-loop structures (SL1 to SL4) [22,23,87,91]. SL1 is the previously mentioned dimerization initiation site, SL2 contains the major splice donor site, and SL3 has been historically considered as the main packaging element [22,23,92]. SL4 contains the AUG start codon, but is fairly unstable and is commonly shown in an alternative base-pairing with the U5 region [12,15,16].

Early attempts to map the HIV-1 packaging signal using deletion mutants identified SL3 as the major packaging signal for HIV-1 [22,23,91]. However, the region SL1–SL4 is not sufficient to direct heterologous RNA into HIV-1 virions [93], and efficient packaging usually requires additional upstream and downstream sequences. Indeed, later efforts to determine the smallest segment of the genome that is capable of packaging heterologous RNA led to conflicting results, with TAR [94], the poly-A stem loop [95], PBS domain [96], SL1 [75,84,96,97], and the first nucleotides of Gag [20,51] all shown to contribute to RNA packaging. Some of these conflicting data likely result from the fact that early deletion studies did not take into account the secondary and tertiary structure of the RNA. For instance, deletions of SL3 preserving the RNA fold only moderately affect packaging [87,91], whereas deletions destroying it decrease packaging by a 100-fold or more [23]. Indeed, a study using targeted deletions of SL1 to SL4 found that deletion of SL3 only reduces the packaging efficiency of the gRNA by two-fold, whereas deletion of SL1 reduces it by five-fold [76]. This points to SL1 rather than SL3 as the main packaging determinant of the gRNA.

It is assumed that HIV-1 packaging is mainly driven by a specific interaction between Gag and the gRNA. Until recent years many in vitro studies of the Gag–gRNA interaction were performed with GagΔp6 for ease of production and purification. However, the p6 domain is known to have an important role in viral assembly [98,99,100] and it is possible that GagΔp6 does not fully recapitulate RNA binding by the full length Pr55^Gag^ precursor protein. Recently, Gag including the p6 domain was successfully purified [101], and extensively tested for binding in vitro to a large panel of gRNA mutants by biochemical and footprinting assays [13]. In agreement with packaging assays conducted in cells, deletion of SL1 had a more drastic effect on full length Gag binding in vitro compared to deletions in SL3. More importantly, footprinting and mutagenesis experiments clearly identified the basal part and internal loop of SL1 as the primary Gag binding site. This was confirmed using mutational interference mapping experiment (MIME), which is a ‘functional footprinting’ technique that can identify at single-nucleotide resolution RNA sequence and structure crucial for function [68]. MIME precisely defined a 110 nucleotide region (positions 227-337 in the NL43 strain) critical for full length Gag binding in vitro (Figure 2B), and quantitatively demonstrated that SL1 is more important for binding than SL3. These data are consistent with NC binding sites identified within the HIV-1 virus by chemical probing [102], and with the crosslinking immunoprecipitation with high-throughput sequencing (CLIP-seq) data of RNA bound to Gag in transfected cells [57].

A recent analysis combining photo crosslinking with selective 2′-hydroxyl acylation analyzed by primer extension (XL-SHAPE), using Gag∆p6 identified multiple binding sites within the HIV-1 5′ UTR, including the poly-A loop, a GU-rich region around nucleotide 120, the PBS domain, several sites within SL1, the internal loop of SL2, the apical loop of SL3, and regions flanking SL3 and the AUG start codon [103]. However, Gag–gRNA binding specificity has been shown to change during virion biogenesis [57], consequently, these additional sites may reflect different binding specificity for RNA of Gag∆p6 compared to full length Gag, or alternatively these additional binding sites may be due to the non-competitive conditions used in this study. Other regions of the HIV-1 5′ UTR are reported to be important for genome packaging, such as TAR [104,105], the poly-A stem loop [106] and the PBS domain [96], as well as distal RNA elements such as a region overlapping the Gag–Pol ribosomal frameshift signal [24] (Figure 2C) and the RRE (Figure 2D) [25,26]. Nevertheless, the role of these distal elements in genome packaging is still under debate, as RNA genomes containing mutations or deletions of the frameshift element [107], or containing the MPMV CTE instead of the RRE are nevertheless efficiently packaged into virions [108]. Whether these additional packaging signals are Gag–gRNA interaction sites, or reflect alternative mechanisms necessary for packaging, such as RNA localization or translational read-through, is not currently known.

### 4.3. Discrimination between Spliced and Genomic RNA, and the Regulation of Gag Binding

**Figure 3:**
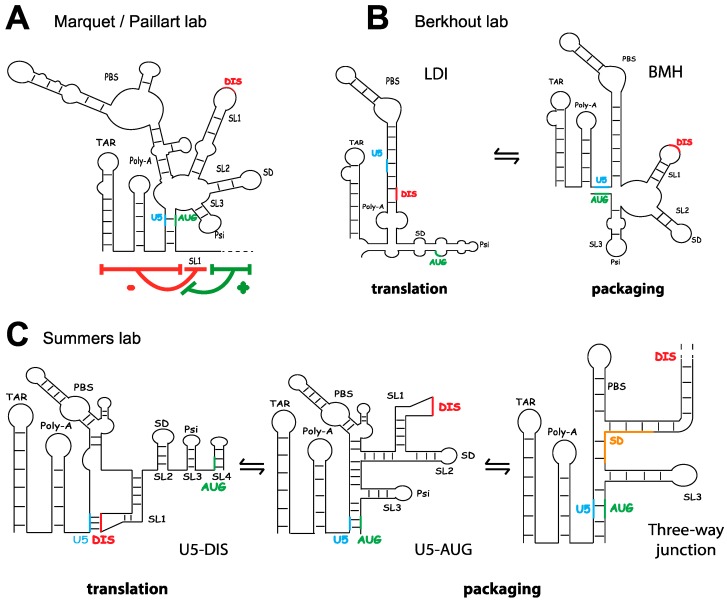
RNA structural models proposed to regulate the packaging and translation of the gRNA. (**A**) Gag binding is subjected to a mechanism of double regulation. The region upstream of SL1 (present in all viral RNAs) negatively regulates Gag binding. A region upstream of SL1 prevents binding of Gag to SL1 and this negative effect is counteracted by a motif downstream of SL4, present only in gRNA species; (**B**) The Long Distance Interaction (LDI) proposed to promote translation and the Branched Multiple Hairpin (BMH) proposed to promote packaging; (**C**) The U5 region is involved in alternative interactions with the dimerization initiation site (DIS) (promoting translation) and the region surrounding the AUG start codon (promoting packaging). A three-way junction, which further extends the U5-AUG interaction and eliminates SL2, is proposed to favour packaging. TAR: Tat responsive element; Poly-A: polyadenylation signal, U5: unique 5′ region, PBS: primer binding site; SL: stem loop; DIS: dimerization initiation site; SD: splice donor; Psi: packaging signal; AUG; start codon.

HIV-1 packaging of the gRNA dimer is strongly favored over abundant cellular RNAs and viral spliced mRNA species. As previously described, the specific recognition motif for the Gag precursor was identified as the internal loop and distal helix of SL1 [13,68]. These results were unexpected as SL1 is located 5′ of the spliced donor site and is thus present both on the genomic and on the spliced viral RNAs, suggesting a mechanism preventing Gag binding to SL1 in spliced viral RNA. Consistent with this idea, SL1 promotes exclusively the packaging of the unspliced gRNA, whereas spliced viral RNAs deleted for SL1 are incorporated with equal efficiency as spliced RNAs containing SL1 [76]. It was proposed that binding of the Gag precursor is controlled through a mechanism of double regulation: the region 5′ of SL1, present on all viral RNAs, prevents binding of Gag to SL1, whilst a downstream region in the Gag gene, only present in the gRNA, abolishes this negative effect [13] (Figure 3A). This mechanism probably requires tertiary interactions that cooperate to build a high affinity Gag binding site, but these remain to be identified.

Given that the dimerization signal overlaps with the packaging signal, it is possible that Gag recognizes the gRNA dimer rather than two monomers. Evidence for this is provided by the observation that the duplication of the HIV-1 packaging site in one full length RNA leads to the packaging of the monomer with an intramolecular interaction [109]. Indeed, the dimerization and encapsidation of HIV-1 gRNA seem to be strongly interrelated processes [76,84,97,110,111,112,113,114], and this feature is observed in all retroviruses studied to date [9,18,115,116,117]. This has given rise to several models where conformational changes in the gRNA regulate Gag binding through dimerization. The first model proposed a switch between the Branched Multiple Hairpins (BMH) and the Long Distance Interaction (LDI) structure [12,118] (Figure 3B). In the LDI, SL1 and poly-A base pair to form a long helix which sequesters, and renders inaccessible for dimerization, the DIS sequence. Conversely, the Gag start codon (AUG) is located in a bulge and proposed to be available for the RNA translation. In the BMH, SL1 to SL3 form stem-loop structures, and the AUG is found base-paired with Unique-5′ region (U5), forming the so-called U5–AUG interaction. This conformation exposes SL1 to promote dimerization and packaging rather than translation [119]. Despite the attractiveness of this model, the LDI conformation has not been detected in cells [88,102], thus rendering any role of this conformation in the packaging of the gRNA very unlikely. On the contrary, the existence of the U5–AUG interaction is well supported by phylogenetic, biochemical and chemical probing data [12,102,120], and is reported to be conserved in HIV-2 [121]. Nuclear magnetic resonance (NMR) structural data showed that the U5–AUG interaction exists in equilibrium with an alternative interaction between the U5 region and the GC-rich loop of SL1 [16] (Figure 3C). In this model, the U5–AUG interaction promotes RNA dimerization and packaging through release of the DIS [122]. A more recent NMR study performed on a 155-nucleotide region of the HIV-1 RNA, comprising nucleotides 1-344, deleted for TAR, Poly-A and PBS, revealed the existence of a tandem three-way junction structure [15] (Figure 3C). This structure extends the U5–AUG interaction to include residues of the major splice donor site in SL2, whilst exposing unpaired and mismatched G residues thought to favour NCp7 binding [15]. Stabilising the U5–AUG interaction stimulates recovery of RNA dimers isolated from cells [123], and mutations to the region surrounding the Gag AUG start codon inhibit packaging [15,16,51]. Furthermore, biophysical measurements show that disrupting the U5–AUG interaction decreases binding of the mature NCp7 protein [15]. However, not all mutations to SL4 predicted to disrupt the U5–AUG interaction affect binding of the full length Gag precursor protein in vitro [13,68], and mutations stabilising SL2 but disrupting the three-way junction surprisingly enhanced Gag binding in one in vitro study [68]. One possible interpretation is that the U5–AUG interaction is not involved in the initial recognition of the gRNA by the Gag precursor protein, but acts at a later stage of packaging after NCp7 is processed.

### 4.4. The Role of Cellular RNAs

Up to 50% of the HIV-1 packaged RNAs are from the cellular host [93,124], including 7SL RNAs, tRNAs and ribosomal RNAs (rRNAs) [124,125,126,127]. Whilst the packaging of spliced viral RNAs has been shown to be specific, and competes with the gRNA for binding to Gag, cellular RNAs are packaged via different and uncertain mechanism(s) [76], and their precise role in the virus life-cycle remain unclear (reviewed in [128,129]).

RNA is known to aid viral assembly by promoting Gag oligomerization [62,130,131], but also regulates binding of the Gag precursor to cellular membranes. The MA domain at the N terminus of HIV-1 Gag is positively charged, and can interact with RNAs and cellular lipids, such as PIP_2_ [54,55]. Intriguingly, the MA–RNA and PIP_2_ binding sites overlap, giving rise to the idea that cellular RNA directs MA to the appropriate cellular membrane [55,56,57]. Strikingly, CLIP-seq experiments performed in cells revealed that the basic region of MA binds to a subset of tRNAs [57]. Thus, HIV-1 Gag uses cellular tRNAs to regulate membrane binding, probably to prevent non-productive intracellular assembly.

## 5. Where Does the Gag–RNA Interaction Occur?

Imaging techniques have the potential to directly visualise Gag–RNA interactions at the scale of individual molecules in real time. Recent advances have begun to shed light on viral assembly from the initial selection of the gRNA by Gag to virus particle assembly at the plasma membrane [132].

In respiratory syncytial virus (RSV), a simple retrovirus, a small proportion of Gag traffics into the nucleus, and this was reported to be required for the efficient encapsidation of unspliced genomes [133,134]. Within the nucleus, RSV Gag binding to the gRNA stimulates its association with the CRM1/RanGTP export complex, leading to its export and packaging [135,136]. Nuclear trafficking is dependent on the MA domain, and is undetectable in an RSV chimera containing HIV-1 MA [137]. Interestingly, this HIV-RSV chimera was still able to generate infectious virus, indicating that nuclear trafficking may not be absolutely required, or alternatively that low levels of residual shuttling may be sufficient for genome packaging [137]. In HIV-1, unspliced RNA is exported from the nucleus using the Rev protein, abrogating the need for Gag trafficking [136]. Nevertheless, the HIV-1 NC domain has been reported to contain a nuclear localization signal that can localize Gag to the nucleoli [138,139], but the functional significance of this is unclear and controversial [137,140].

For HIV-1, the initial Gag–gRNA interaction is thought to occur in the cytoplasm [131,141,142,143,144]. Genetic evidence, using a recombination–based assay, clearly shows that encapsidation occurs after RNA export from the nucleus [82,108]. However, attempts to image the first interaction site within cells have led to conflicting results, with one study identifying this site as the perinuclear/centrosomal region [145], whilst most other studies failed to observe a specific subcellular localization [131,141,143]. Within the cell, biochemical and imaging studies identify two different oligomerization states of Gag, depending on the timing of viral assembly and release: as a monomer or low-order oligomers in the cytosol [131], and high order multimers found preferentially at the plasma membrane [57,131,144]. Cytosolic oligomerisation depends on the NC domain, indicating a role for RNA in this process [131], but is independent of the ability of Gag to bind to membranes [57,144]. Interestingly, it has been observed that the Gag precursor protein is likely a trimer in solution [13], and in vitro assays showed that about six Gag molecules associate with one gRNA molecule with high affinity [146]. Taken together, these data support the notion that gRNA selection initiates in the cytoplasm and involves a very limited number of Gag molecules, which are then transported to the plasma membrane for assembly.

## 6. Transport of the Gag–RNA Complexes to Assembly Sites

A subcomplex comprising the gRNA and a few Gag proteins must traffic through the cytoplasm to sites of assembly. A dense network of actin filaments underneath the plasma membrane is visualized at HIV-1 budding sites, and viral particles have been shown to contain substantial amounts of actin [147], but its involvement in the assembly process has not been clearly demonstrated [148,149,150]. Live cell imaging by fluorescence microscopy and single-molecule tracking have revealed that in the absence of Gag, the HIV-1 gRNA moves randomly within the cell, indicating that it is not actively transported through the cytoplasm [42]. Furthermore, treatment of cells with cytochalasin-D and nocodazole demonstrate that an intact cytoskeletal structure is not required for HIV-1 RNA trafficking, consistent with diffusion as the major transport mechanism [42]. Many cellular proteins, such as ATP binding cassette subfamily E member 1 (ABCE1) [151,152], Staufen1 [153] and DEAD-box helicase 6 (DDX6) [154], traffic with the Gag–gRNA complexes to the plasma membrane. Staufen-1 binds the gRNA and is thought to be involved in packaging [153,155], but in general, the mechanism, purpose and path of these cellular-Gag–gRNA complexes remain poorly understood [156].

Reports initially supported a “late endosomal” model for HIV-1 assembly based on the fact that a significant proportion of Gag was found to localize in cellular compartments containing endosomal markers, like CD63 and lysosome-associated membrane protein-1 (Lamp-1) [157,158,159]. However, it was later realized that visualization of CD63-positive invaginations of the plasma membrane by electron microscopy could be mistaken for intracellular compartments containing viruses [149,160]. This hypothesis was confirmed by using two pharmacological inhibitors that abolish late endosome motility without affecting the amount of released particles [149]. Immunofluorescence studies also showed that Gag is first targeted to the plasma membrane and thereafter a population of virions can subsequently be internalized in late endosomes [149]. Taken together, these data are not consistent with HIV-1 endosomal trafficking pathway.

Total internal reflection fluorescence (TIRF) microscopy is an imaging technique that can visualize the Gag–gRNA interaction at the plasma membrane [110,161,162]. TIRF studies show that in the absence of Gag, gRNA only appears transiently at the membrane [161,162], whereas in the presence of Gag, the gRNA is able to anchor at the membrane in a packaging signal dependent manner [110,143,149]. Anchoring is invariably followed by the rapid accumulation of Gag and the formation of a viral particle [110,161]. RNA dimerization is important for Gag binding and genome packaging, but it is unknown whether genome dimerization occurs before Gag binding and trafficking to the plasma membrane. One of the early TIRF studies proposed that preformed gRNA dimers are recruited to sites of assembly, since the RNA fluorescence signal never increased once at the plasma membrane together with Gag [161]. However, this picture has been revised based on a two colour TIRF imaging system that can directly visualize RNA dimerization at sites of viral assembly [110]. Surprisingly, dimerization was frequently seen to occur at the membrane, rather than being recruited preformed from the cytoplasm, and in a few cases RNA dimerization could still be observed at late stages of assembly [110]. Importantly, however, a recent two colour super-resolution microscopy study observed significant levels of RNA dimerization already in the cytoplasm, albeit with higher levels at the plasma membrane [141]. One possible way to reconcile these observations is to consider that Gag initiates RNA dimerization and/or stabilizes gRNA dimers in the cytoplasm, but that RNA dimerization remains dynamic at the membrane until virus assembly is completed [110,141].

## 7. Maturation

After assembly and the release from host cells, virions undergo a process of maturation leading to morphological rearrangements of the particle with the formation of the core and increased stability of the RNA dimer [3]. The proteolytic processing of the Pr55^Gag^ and Pr160^Gag–Pol^ precursors, performed by the viral protease, allows the production of the structural proteins and is required for the morphological rearrangement of the viral particle [163]. This process is believed to happen during or quickly following budding of the immature particle [164]. Proteolytic processing is highly regulated temporally as protease inhibitor wash-out experiments clearly demonstrate that the timing of proteolytic processing is critical for the acquisition of infectivity [165], and viral infectivity is blocked even at concentrations of inhibitor showing little to no effect on Gag processing [166,167]. Concomitant with proteolytic processing the gRNA is rearranged from a “loose” unstable dimer to a “tight” dimer that has slower electrophoretic mobility and altered thermal stability compared to dimers isolated from immature viruses [80,168].

The maturation process is crucial for the acquisition of viral infectivity and multiple lines of evidence indicate a link between proteolytic processing and the structural maturation of the gRNA. For Moloney murine leukemia virus (MuLV), immature genomes isolated from protease negative virions adopt an unstable conformation with fewer intergenome interactions when compared to mature virus [169]. RNA genomes isolated from protease defective HIV-1 are unstable, much like RNA dimers isolated from immature viruses [170,171]. The production of the different structural proteins therefore seems important for the structural maturation of the gRNA. The NCp7 protein is well-known for its nucleic-acid chaperone activity and is the leading candidate for causing RNA dimer stabilization [172,173,174]. Cryo-electron tomography and subtomogram averaging highlighted the importance of the primary cleavage between p2 and NC domains of Gag to initiate dimer RNA stabilization, while the ensuing cleavages are necessary to complete the process [175]. This change in NC RNA chaperone activity during proteolytic processing [60,176,177,178] is likely to regulate reverse transcription through multiple mechanisms including the promotion of stable tRNA annealing [179,180], facilitation of strand transfer [181,182,183], regulation of reverse transcription initiation [184,185,186], and the general remodelling of RNA structure, possibly to prevent reverse transcriptase stalling [187,188]. Recently, the impact of the RNA genome on proteolytic processing has also started to be evaluated. The analysis of the proteolytic processing pattern of HIV-1 [189] and HIV-2 [190] dimerization mutants indicates an accumulation of the p41 (MA-CA-p2) intermediate. Dimerization and the RNA genome thus could have an impact on particle assembly by modulating Gag processing [173].

## 8. Conclusions

The selection of the gRNA from the cytoplasm and its recruitment into budding virions by Gag is a remarkable process, and recent studies have only begun to reveal its complexity. Although tremendous progress has been made, clearly our understanding is far from being complete. Important questions remain regarding the selection of the full-length gRNA by Gag, and in particular the mechanistic details of how this process is regulated. From the viewpoint of the RNA genome, there are several proposed structural models of the 5′ UTR that are yet to be integrated, which point towards a conformational flexibility that is most certainly involved in the regulation of the viral life cycle. Further advances in RNA structural analysis are likely needed to resolve (1) how Gag selects between genomic and spliced viral RNAs; (2) how RNA structural switches control translation versus packaging; and (3) the location of long hypothesized dimerization sites outside of SL1. From the viewpoint of the Gag protein, most binding studies have been performed using unmyristoylated GagΔp6, which may not completely reflect the properties of myristoylated full length Gag found in cells. Given the importance of both the myristoyl group and the p6 domain to virus assembly, it is tempting to speculate that these features could impact the Gag–gRNA interaction. Finally, a major limitation of existing imaging techniques is their inability to detect low numbers of Gag molecules, and this has so far prevented the unambiguous determination of where and when Gag first interacts with the gRNA. Improved imaging technologies are sure to address this problem in the near future.
